# Myasthenia Gravis Presenting With Vocal Cord Paralysis As the Initial Symptom Occurring One Week Following Herpes Zoster Vaccination: A Case Report and Review of the Literature

**DOI:** 10.7759/cureus.83454

**Published:** 2025-05-04

**Authors:** Shimpei Asada, Hikaru Odera, Koji Morishita, Shusuke Mori

**Affiliations:** 1 Department of Critical Care and Emergency Medicine, Tokyo Women's Medical University, Tokyo, JPN; 2 Department of Acute Critical Care and Disaster Medicine, Institute of Science Tokyo, Tokyo, JPN

**Keywords:** inactivated herpes zoster vaccine, laryngeal type, myasthenia gravis, shingrix, side effect post vaccination, vaccination reaction, vocal cord paralysis

## Abstract

Myasthenia gravis (MG) is an autoimmune neuromuscular junction disorder primarily characterized by fluctuating skeletal muscle weakness. While it typically presents with ocular or generalized symptoms, vocal cord paralysis (VCP) as an initial manifestation is exceedingly rare and often leads to diagnostic delays. We report the case of an 86-year-old Japanese woman who developed progressive hoarseness, dysphagia, and respiratory distress approximately one week after receiving the inactivated herpes zoster vaccine (Shingrix®). Initial evaluation revealed unilateral VCP, which progressed to bilateral paralysis, requiring emergency intubation and subsequent tracheostomy. Despite extensive imaging and laboratory investigations, the etiology remained unclear until the patient developed diplopia and ptosis around hospital day 25. Although both anti-acetylcholine receptor (AChR) and anti-muscle-specific kinase (MuSK) antibodies were negative, the diagnosis of MG was confirmed through positive edrophonium and ice pack tests. Treatment included pyridostigmine, plasma exchange, and immunosuppressive therapy with tacrolimus and prednisolone. While ptosis improved, bilateral VCP and diplopia persisted at one year, and the patient remained tracheostomy-dependent but functionally independent. This case adds to the limited literature on laryngeal-type MG, a focal subtype marked by bulbar symptoms and often negative for AChR antibodies. Our review of post-2000 literature identified 13 cases of MG with VCP, 10 of which presented with VCP initially. Notably, nine required airway management, underscoring the potential for rapid respiratory compromise. This is the first reported case of MG development temporally associated with herpes zoster vaccination. Although a definitive causal link cannot be established, the close temporal sequence - fever and systemic immune activation shortly after vaccination, followed by MG onset - raises the possibility of an immune-mediated trigger. Previous reports have described MG onset after various other vaccines, including HPV, HBV, influenza, and COVID-19, suggesting that vaccination may rarely unmask or precipitate MG in susceptible individuals. Nonetheless, large-scale studies have not demonstrated a significant increase in autoimmune disease following vaccination, supporting the overall safety of immunization programs. This case highlights the importance of recognizing atypical MG presentations such as isolated VCP, particularly in the context of recent immune stimulation. Early diagnosis and appropriate treatment are critical for improving outcomes and preventing life-threatening complications such as airway obstruction. Heightened clinical suspicion is warranted in similar scenarios to facilitate timely intervention.

## Introduction

Myasthenia gravis (MG) is an autoimmune disorder characterized by dysfunction at the neuromuscular junction - the synapse between motor neurons and muscle fibers. The primary pathological mechanism involves autoantibodies targeting acetylcholine receptors (AChR) or muscle-specific kinase (MuSK), impairing signal transmission. Clinically, MG typically presents with fluctuating skeletal muscle weakness, most commonly manifesting as diplopia, ptosis, bulbar weakness, and generalized fatigue. In severe cases, the disease can rapidly progress to respiratory compromise, known as myasthenic crisis [1]. MG diagnosis relies on a combination of serologic, pharmacologic, and electrophysiologic testing. Detection of anti-AChR or anti-MuSK antibodies supports diagnosis in the majority of cases, although a subset remains seronegative. Pharmacological tests, such as the edrophonium (Tensilon) and ice pack tests, offer rapid bedside diagnostic clues, particularly in ocular presentations. Repetitive nerve stimulation (RNS) and single-fiber electromyography (SFEMG) are instrumental for neurophysiological confirmation, especially in seronegative MG.

Vocal cord paralysis (VCP), by contrast, has a broad differential diagnosis. Common etiologies include recurrent laryngeal nerve injury (e.g., post-surgical or neoplastic compression), viral neuropathy, idiopathic causes, and neurodegenerative disorders. In rare instances, MG can present with isolated laryngeal involvement. Laryngeal muscle dysfunction in MG may reflect the selective vulnerability of striated laryngeal muscles innervated by cranial nerves, particularly the recurrent laryngeal branch of the vagus nerve. Such focal involvement underscores the heterogeneous and unpredictable clinical spectrum of MG. Understanding this neuromuscular vulnerability helps contextualize how MG could plausibly present with isolated vocal symptoms prior to the emergence of generalized or ocular features.

Although thymoma, autoimmune comorbidities, and infections are well-recognized triggers of MG, recent studies have also suggested a possible association with vaccination, including reports following administration of human papillomavirus and influenza vaccines [2-4]. VCP as the predominant initial symptom of MG remains exceptionally rare, with only a handful of cases reported in the literature, including instances of bilateral vocal fold involvement necessitating airway management [5]. This clinical pattern underscores the diagnostic challenge and potential severity of laryngeal MG, particularly in seronegative or atypically presenting cases. The present report contributes to this limited body of literature by describing a temporally associated onset of MG following herpes zoster vaccination, warranting further attention to this possible, though rare, association.

## Case presentation

The patient was an 86-year-old Japanese woman with no remarkable medical history and previously good functional status without impairment. Fifty-one days prior to admission, she received an inactivated herpes zoster vaccine (Shingrix® intramuscular injection), after which she developed a fever of 38°C the following day. Apart from fever, she exhibited no other accompanying symptoms, and the fever resolved after seven days. However, around the time her temperature normalized, she began to experience episodes of choking, followed by a sense of progressive dysphagia and hoarseness over several days. She consulted an otolaryngologist 20 days post-vaccination, where a laryngeal fiberoptic examination revealed right-sided unilateral VCP. The cause remained unclear, and the decision was made to observe the patient’s condition.

**Figure 1:**
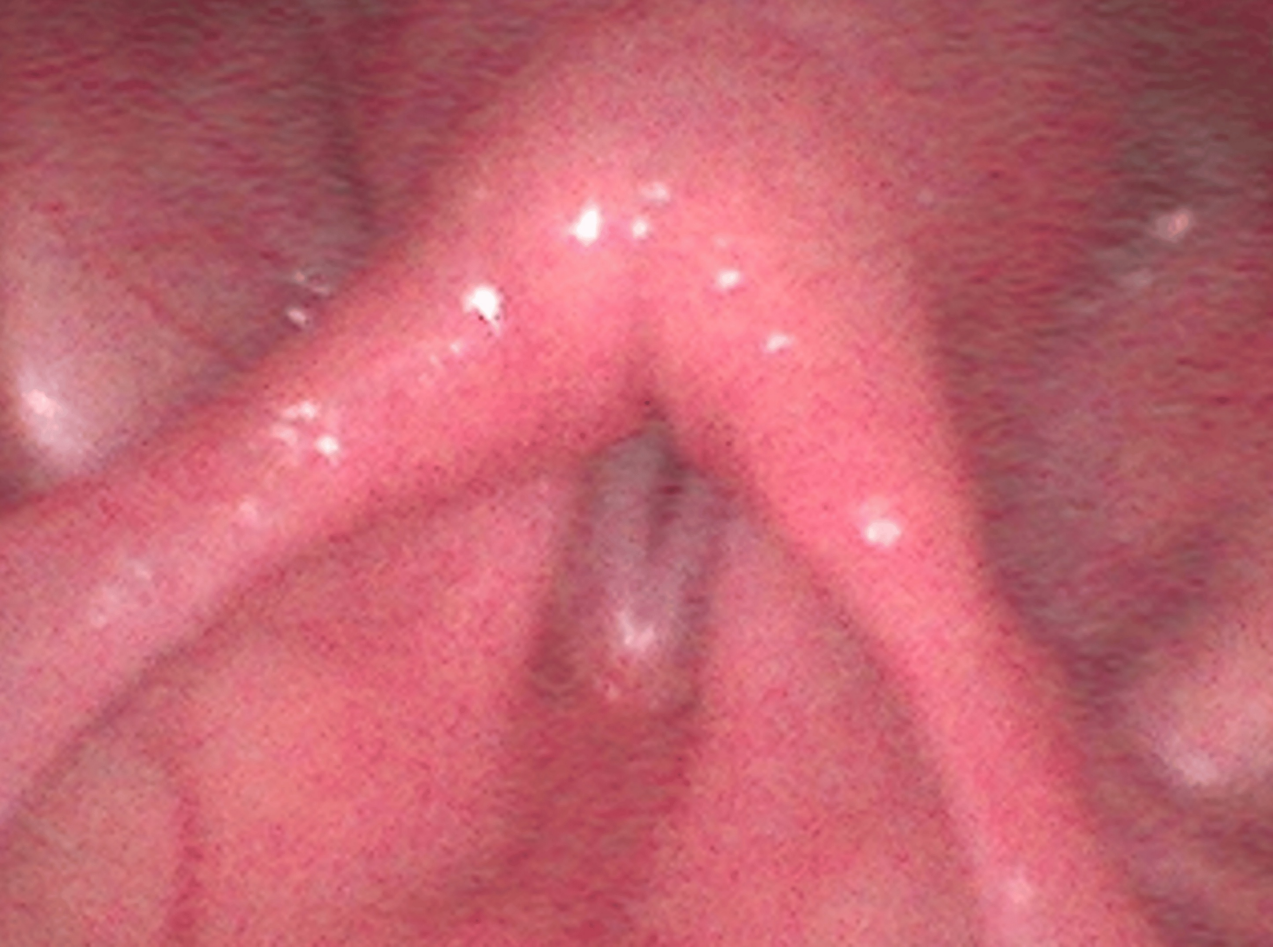
Laryngeal fiberoptic examination showed bilateral vocal cords fixed in a paramedian position. Laryngoscopic findings on admission showed bilateral vocal cords fixed in the paramedian position. The patient, an 86-year-old woman with no significant medical history, developed fever, dysphagia, and hoarseness following administration of Shingrix®, a recombinant inactivated herpes zoster vaccine. Initial laryngoscopy on day 20 post-vaccination revealed right VCP, which progressed to bilateral involvement and severe respiratory distress by the time of admission.

Symptoms continued to progress, and two days before hospital admission, she developed increasing respiratory distress with significant labored breathing, prompting emergency transportation to our facility. Upon arrival, she had a respiratory rate of 30 breaths per minute and SpO₂ of 93%, with marked labored breathing. Physical examination revealed severe hoarseness but no other significant abnormalities. No muscle weakness in the extremities, ptosis, or other neurological deficits were observed, and laboratory tests provided no diagnostic clues. Laryngeal fiberoptic examination showed bilateral vocal cords fixed in a paramedian position (Figure 1).

The patient was diagnosed with respiratory failure due to bilateral VCP, necessitating emergency intubation, after which her respiratory status stabilized.

Head, neck, and chest CT scans, as well as a head MRI, revealed no abnormalities, including the absence of thymoma. Despite inpatient management, the condition did not significantly improve, and tracheostomy was performed seven days later. Repeat fiberoptic examination confirmed persistent bilateral vocal cord fixation in the paramedian position. Her respiratory status remained stable, allowing mechanical ventilation withdrawal by the 10th day of hospitalization, yet the underlying cause remained unknown. The patient did not undergo assessment of Vital Capacity or Negative Inspiratory Force (NIF) because she exhibited no clinical signs of respiratory compromise suggestive of respiratory muscle involvement. On day 31 of hospitalization, the patient reported that she had gradually developed diplopia and ptosis around day 25 of her admission. Retrospective review of her clinical course also suggested fatigability of bulbar symptoms, particularly worsening hoarseness and dysphagia with prolonged vocal effort. Although anti-AChR and anti-MuSK antibodies tested negative, edrophonium (Tensilon) and ice pack tests were positive, leading to the diagnosis of MG; notably, the positive Tensilon test referred to observable clinical improvement, including transient resolution of ptosis and enhanced ocular motility following edrophonium administration. Treatment with pyridostigmine bromide 180 mg/day was initiated, followed by plasma exchange therapy twice weekly for three weeks. Additional immunosuppressive treatment included tacrolimus 2 mg/day and prednisolone 15 mg/day. Two months after initiating therapy, her ptosis improved; however, diplopia and VCP persisted, and she was discharged home with a tracheostomy.

**Figure 2:**
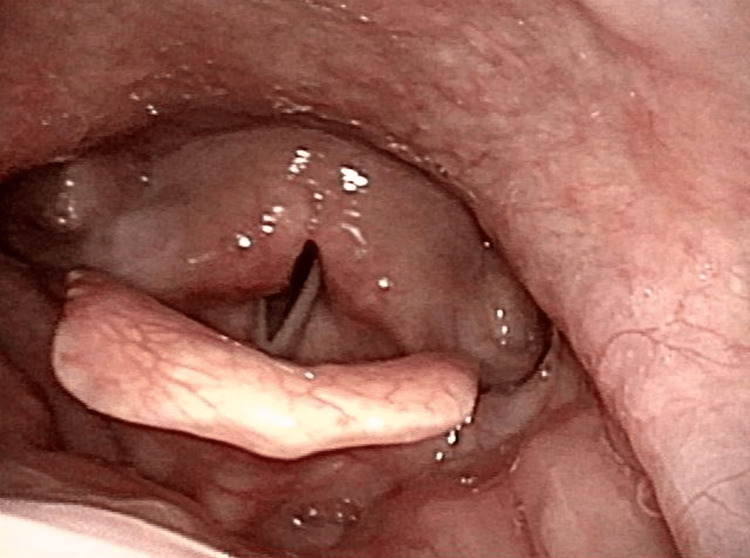
Five months post-discharge, laryngeal fiberoptic examination demonstrated a slight improvement in vocal cord mobility. Laryngoscopy five months post-discharge showed slight improvement in bilateral vocal cord mobility. The patient had initially presented with respiratory failure due to bilateral VCP, requiring intubation and tracheostomy. MG was diagnosed based on positive edrophonium and ice pack tests. Despite immunotherapy, VCP persisted at discharge, with gradual improvement noted during follow-up.

Five months post-discharge, a slight improvement in vocal cord mobility was observed (Figure 2).

However, VCP persisted one year later, making decannulation difficult. Diplopia also persisted, although no further progression of the condition was noted, and she continued to maintain an independent lifestyle.

## Discussion

Isolated VCP as the first manifestation of MG is exceedingly rare. In one large series of 1,520 MG patients, only seven cases (∼0.4%) initially presented with dysphonia due to vocal cord involvement [6]. Our review of the literature since 2000 identified 13 additional reported cases of MG with VCP (Table 1), of which 10 had VCP as the initial symptom [5,7-18].

**Table 1:** Review of the literature since 2000 regarding cases of myasthenia gravis with vocal cord paralysis. AChR: anti-acetylcholine receptor; MuSK: muscle-specific kinase; IVIG: intravenous immunoglobulin; PLEX: plasma exchange Isolated VCP as the first manifestation of MG is exceedingly rare. Our review of the literature since 2000 identified 13 additional reported cases of MG with VCP, of which 10 had VCP as the initial symptom. These patients were evenly split by sex and ranged from 45 to 88 years old, with a median of 69.5 years. In 9 of 11 cases, including the present case, the laryngeal paralysis was severe enough to require endotracheal intubation and mechanical ventilation.

Year	Author	Sex	Age	Previous MG Diagnosis	Trigger	Diagnostic Tests	Thymoma	Associated Symptoms	Intubation	Treatment	Prognosis
2000	Cridge et al [7]	M	88	None	Not reported	Anti-AChR/MuSK negative	(-)	None	Yes	Not reported	Not improved
2002	Teramoto et al. [8]	F	82	14 years	Not reported	Anti-AChR/MuSK negative	(-)	None	Yes	Prednisolone, pyridostigmine	Improved
2007	Hara et al. [9]	M	56	None	Not reported	Anti-MuSK positive	(-)	Dysphagia, paralysis, hoarseness	Yes	IVIG, prednisolone, tacrolimus	Not improved
2007	Kanemaru et al. [10]	M	76	None	Not reported	Anti-AChR positive	(-)	None	Yes	Prednisolone, cyclosporine	Not reported
2008	Sylva et al. [11]	F	24	2 years	Not reported	Anti-MuSK positive	Not reported	Paralysis, stridor	No	Prednisolone, pyridostigmine	Not reported
2010	Khan et al. [12]	M	71	None	Not reported	Anti-AChR positive	(-)	SOB, dysphagia	Yes	Not reported	Improved
2011	Kitagawa et al. [13]	F	86	5 years	Not reported	Anti-AChR positive	(-)	None	Yes	Methylprednisolone	Deceased
2011	Sethi et al. [14]	M	68	None	Not reported	Anti-AChR/MuSK negative	(-)	None	Yes	Neostigmine	Not reported
2014	Sasaki et al. [15]	F	78	None	Not reported	Anti-AChR positive	(+)	Dysphagia	Yes	Immunosuppressive, thymectomy	Not reported
2020	Balabbigari et al. [16]	F	51	None	Not reported	Anti-AChR/MuSK negative	(-)	Hoarseness	Yes	Prednisone, mycophenolate	Not reported
2020	Nelke et al. [5]	F	80	None	Not reported	Anti-AChR positive	(+)	Dysphagia	Yes	Plasmapheresis, corticosteroids	Deceased
2021	Santilli and Stitt [17]	F	45	None	Not reported	Anti-MuSK positive	(-)	Hoarseness	No	PLEX, rituximab	Not reported
2022	Beka et al. [18]	F	58	None	Upper airway infection	Anti-AChR positive	(+)	None	No	Corticosteroid	Not reported
2023	Our case	F	86	None	Vaccination	Anti-AChR/MuSK negative	(-)	Dysphagia, hoarseness	Yes	Prednisolone, pyridostigmine	Not improved

These patients were evenly split by sex and ranged from 45 to 88 years old (median 69.5 years). In 9 of 11 cases, including the present case, bilateral VCP required endotracheal intubation and mechanical ventilation, underscoring the potential for life-threatening respiratory failure.

These cases likely represent a distinct MG subtype -“laryngeal-type” MG-characterized by early bulbar symptoms such as dysphonia, dysphagia, and dysarthria, with minimal or absent ocular involvement. Similar to ocular MG, this subtype shows a lower seropositivity rate for acetylcholine receptor (AChR) antibodies (~45%) compared to generalized MG (~80-90%) [1,19]. Our literature review found a comparable seropositivity rate of 45% among VCP-predominant cases, reinforcing this observation.

Diagnosing laryngeal-onset MG remains challenging due to its atypical presentation, which may mimic primary ENT disorders. Yang X et al. [20] reported that only ~23% of patients were diagnosed with MG at their initial evaluation. Diagnosis is often delayed until classical ocular symptoms, such as ptosis or diplopia, develop. Clinician awareness is essential to facilitate early recognition and prevent respiratory compromise.

Recent studies have improved our understanding of seronegative laryngeal MG. Mullen et al. highlighted key features such as frequent antibody negativity, diagnostic utility of the edrophonium (Tensilon) test, and responsiveness to pyridostigmine [21]. Electrophysiological tests like laryngeal electromyography (EMG) and repetitive nerve stimulation (RNS) were particularly sensitive in these patients. Incorporating these tools is critical in antibody-negative cases.

The Tensilon test has a reported sensitivity of 71-95% and specificity of 97-100% [22,23]. The ice pack test, primarily used for ptosis, has a sensitivity of 80-90% and nearly 100% specificity [23,24]. Though less validated in laryngeal MG, it remains a useful bedside tool when positive.

Electrodiagnostic testing remains a cornerstone of MG diagnosis. RNS detects decremental responses in 75% of generalized MG, while single-fiber EMG offers the highest sensitivity (85-100%) [23,25]. These tests are indispensable when antibody testing is negative and clinical suspicion remains high.

Review of reported cases reveals variable treatments depending on severity and serostatus. Common therapies include pyridostigmine, corticosteroids, IVIG, plasma exchange, and immunosuppressants such as azathioprine, cyclosporine, or tacrolimus [16,18,20]. Acute airway compromise often requires tracheostomy. Prognosis is heterogeneous - some patients recover fully, while others have persistent bulbar dysfunction or require long-term ventilatory support [7,18]. These outcomes underscore the importance of early, individualized treatment.

The present case is notable for symptom onset shortly after varicella-zoster vaccination. Although several case reports suggest temporal associations between vaccination and MG onset [4,26], large-scale reviews have not demonstrated causality. For example, Mailand MT, Frederiksen JL [27] found no consistent evidence linking vaccines to MG or other autoimmune neurologic disorders. Given the widespread use of vaccines, some MG cases will coincide with recent vaccination purely by chance.

Proposed mechanisms for post-vaccination MG include molecular mimicry and immune dysregulation similar to that seen with infections [28]. In our case, the patient developed bulbar weakness immediately after a post-vaccination fever, suggesting a possible immune trigger. However, given limited evidence for a causal link, this association should be interpreted cautiously.

It is also important to acknowledge the limitations of single case reports. While they can highlight rare presentations and hypotheses, they lack generalizability and cannot establish causality. Prospective studies with larger cohorts are essential to determine whether vaccination can meaningfully contribute to MG onset.

This case reinforces the need for early recognition of laryngeal MG, especially when initial symptoms are restricted to the bulbar musculature. A high index of suspicion, timely diagnostic testing, and individualized treatment can prevent airway compromise and improve patient outcomes.

## Conclusions

The key lesson from this case is not to alarmingly conclude that vaccines cause MG, but rather to maintain a high index of suspicion for neuro-immunological diseases like MG, in the wake of a recent vaccination. Physicians should be aware that MG can present with atypical features (such as isolated laryngeal symptoms) and, on rare occasions, may coincide with or follow immunizations. This awareness can facilitate timely diagnosis and treatment. In a treatable condition such as MG, early recognition and appropriate therapy, whether the disease is ocular, generalized, or a focal laryngeal subtype, are critical for improving patient outcomes. By acknowledging the potential (albeit rare) links between immune triggers (like infections or vaccines) and MG, clinicians will be better prepared to identify unusual presentations and intervene before the disease progresses to a life-threatening stage.
